# The High Immunity Induced by the Virus-Like Particles of Foot-and-Mouth Disease Virus Serotype O

**DOI:** 10.3389/fvets.2021.633706

**Published:** 2021-02-25

**Authors:** Yan Xiao, Suling Zhang, He Yan, Xiaolin Geng, Yanwei Wang, Xin Xu, Mengyue Wang, Haohao Zhang, Baicheng Huang, Wenqiang Pang, Ming Yang, Kegong Tian

**Affiliations:** ^1^College of Animal Science and Veterinary Medicine, Henan Agricultural University, Zhengzhou, China; ^2^National Research Center for Veterinary Medicine, Luoyang, China; ^3^National Centre for Foreign Animal Disease, Winnipeg, MB, Canada

**Keywords:** foot-and-mouth disease virus serotype O, virus-like particles, neutralizing epitopes, humoral immunity, cellular immunity

## Abstract

Foot-and-mouth disease (FMD), caused by FMD virus (FMDV), is a highly contagious and economically devastating viral disease of cloven-hoofed animals worldwide. In this study, the coexpression of small ubiquitin-like modifier (SUMO)–fused capsid proteins of FMDV serotype O by single plasmid in *Escherichia coli* was achieved with an optimal tandem permutation (VP0–VP3–VP1), showing a protein yield close to 1:1:1. After SUMO removal at a low level of protease activity (5 units), the assembled FMDV virus-like particles (VLPs) could expose multiple epitopes and have a size similar to the naive FMDV. Immunization of pigs with the FMDV VLPs could induce FMDV-specific humoral and cellular immune responses effectively, in a dose-dependent manner. These data suggested that the stable FMDV VLPs with multiple epitope exposure were effective for the induction of an immune response in pigs, which laid a foundation for the further development of the FMDV subunit vaccine.

## Introduction

Foot-and-mouth disease (FMD) is a highly contagious and economically devastating viral disease of cloven-hoofed animals (1), which is endemic in many countries including parts of Asia, Africa, South America, and at the periphery of the European Union (2). The disease is caused by the FMD virus (FMDV), which belongs to the genus *Aphthovirus* within the family Picornaviridae. The virus exists as seven distinct serotypes (O, A, C, Asia1, SAT1, SAT2, and SAT3) as well as multiple subtypes that have been generated during the evolution of the virus (3, 4).

The FMDV genome contains a single large open reading frame, which encodes a precursor polyprotein that is processed by virus-code proteases for the formation of structure (VP0, VP1, and VP3) and nonstructural proteins. During virus maturation, capsid protein VP0 is further self-cleaved to capsid proteins VP2 and VP4. VP1, VP2, and VP3 are exposed on the surface of the virus, whereas VP4 is located internally (5, 6).

Numerous subunit vaccines have been developed by utilizing various expression systems with different host cells, including insect cells (7), *Escherichia coli* (8–11), silkworm larvae (12), mammalian cells via recombinant vaccinia virus (5) or transfection (13), transgenic alfalfa plants, tomato fruits (14), and *Nicotiana benthamiana* expression platform (15, 16). Coexpression of P1-2A and 3C protease, which cleaves P1-2A into capsid proteins and then self-assemble to form virus-like particles (VLPs), had been achieved *in vivo* in many expression systems, except in *E. coli*, but the acid sensitivity and thermolability were the main concerns. Recently, two or three plasmids with different antibiotic selection markers were used to obtain the coexpression of FMDV capsid protein as the soluble form in *E. coli*.

The report showed that the expression of three small ubiquitin-like modifier (SUMO)–fused FMDV capsid proteins using two plasmids in *E. coli* showed a ratio of protein yield close to 1:1:1 (VP0:VP1:VP3), and then the tertiary protein complex after cleaving by SUMO protease was able to assemble into VLPs *in vitro* (9). The SUMO-fused protein of FMDV VP0, VP1, and VP3 using three plasmids also achieved in *E. coli* and the immunogenicity of the VLPs were evaluated in cattle (8). However, the expression strategy of multiple vectors may lead to the disappearance of different antibiotic selection markers in cells during cell culture, which may cause the uncoordinated proportion of three FMDV capsid proteins.

Previously, we developed an improved *E. coli* coexpression system by coexpressing SUMO fused full-length FMDV capsid proteins in tandem driven by a single plasmid and the protein yield closed to 50 mg/L (11). However, the effect of the tandem permutation of VP0, VP1, and VP3 in a single plasmid on the proportion of three FMDV capsid proteins and the cleavage rate of the SUMO protease on the assembly efficiency of VLPs have not been thoroughly studied.

In order to obtain the high-quality VLPs, here we optimized the tandem permutation of VP0, VP1, and VP3 in a single plasmid, and the SUMO protease cleavage rate also is evaluated. The uniform proportions of VP0, VP1, and VP3 were efficiently expressed in *E. coli*, as shown by screening six permutations. After the removal of SUMO from the fusion proteins, the VP0–VP1–VP3 complex could be assembled into VLPs, which is affected by the rate of SUMO protease digestion. Furthermore, these VLPs were characterized and verified prior to use as an immunogen in pigs for the immunity evaluation.

## Materials and Methods

### Animals

Pigs with the weight of ~40 kg (*n* = 20) used in this study were tested negative for FMDV serotype O antibodies by a commercial liquid-phase-block enzyme-linked immunosorbent assay (LPB-ELISA) kit (Lanzhou Veterinary Research Institute, China). All the animal samples were collected according to the protocol approved by the Animal Care and Ethics Committee of National Research Center for Veterinary Medicine (Permit 20200825056).

### Monoclonal Antibody

The binding abilities of the VLPs and the unassembled capsid proteins were analyzed by four neutralizing monoclonal antibodies (MAbs) of FMDV (F21-48, F21-64, F21-58, and F21-41) provided by Dr. Yang (National Centre for Foreign Animal Disease, Canada) (17).

### Protein Expression

An improved expression vector of SUMO fusion protein was constructed as described previously (11). The primers used in this study are listed in Supplementary Table 1. Briefly, the full-length VP0, VP3, and VP1 coding regions of FMDV serotype O (GenBank accession no. JQ973889.1) were synthesized (Genewiz) and were cloned into the plasmid pETSUMO, designated as pETSUMO-VP0, pETSUMO-VP3, and pETSUMO-VP1, respectively. For the construction of pET-Mya98-VP013, the DNA fragments of 6his-SUMO-VP0 in the plasmid pETSUMO-VP0 were amplified with primers P1 and P2 and then inserted into vector pET28b (Novagen, USA) to produce pET-Mya98-VP0 after the digestion by *Nco*I and *Nde*I. Subsequently, the VP3 expression cassette in the plasmid pETSUMO-VP3 was amplified with primers P3 and P4, after the digestion by *Bam*HI and *Sac*I, the 6his-SUMO-VP3 was inserted into the pET-Mya98-VP0 for the construction of pET-Mya98-VP03. After that, the last expression cassette 6his-SUMO-VP1, amplified from pETSUMO-VP1 with primers P5 and P6, was inserted into pET-Mya98-VP03 by *Sal*I and *Not*I, and then the plasmid pET-Mya98-VP031 was constructed. The constructions of the rest expression vectors pET-Mya98-VP013, pET-Mya98-VP103, pET-Mya98-VP130, pET-Mya98-VP301, and pET-Mya98-VP310 (Supplementary Figure 1) were according to the strategy of pET-Mya98-VP013 production. The recombinant plasmids were transformed into *E. coli* BL21 (DE3) competent cells (Qiagen, USA), and the expression was carried out as described before (11).

### Protein Purification

The bacteria were harvested by centrifugation, and then the cell pellets were resuspended, disrupted, and purified as previously described (11). The elution samples were analyzed by 12% sodium dodecyl sulfate–polyacrylamide gel electrophoresis (SDS-PAGE). The relative proportion of the three SUMO fusion proteins was analyzed by Alpha Imager 2200. The imidazole was removed from the elution samples with buffer A [20 mM Tris–HCl, 300 mM NaCl (pH 7.0)] by using a 12-kDa dialysis membrane. The SUMO protein tag was removed by SUMO protease digestion and removed by Ni Sepharose 6FF (GE Healthcare, USA).

The recombinant proteins were injected into the AKTA Pure system connected to a HiLoad 16/600 Superdex 200-pg gel filtration column (GE Healthcare, USA) that was pre-equilibrated with gel filtration buffer [20 mM Tris–HCl, 300 mM NaCl (pH 7.0)] with a flow rate of 1.0 mL/min at 25°C. Fractions that eluted under the major monodisperse peak were pooled and were analyzed by 12% SDS-PAGE and dynamic light scattering (DLS). The eluate was monitored by UV280, and the samples were used for the subsequent analysis. DLS was used to characterize the hydrodynamic diameter of proteins and performed using NANO ZSE (Malvern, UK).

### Electron Microscopy

Purified proteins were measured by transmission electron microscope (TEM). A copper grid was dipped with a drop of the proteins at room temperature (RT) and then dried gently using filter paper and stained with 3% phosphotungstic acid for 5 min. The excess liquid was removed with filter paper, and the samples were examined under a TEM at 80 kV (FEI, USA).

### Solution Competitive ELISA

Solution competitive ELISA was used to assess the median inhibitory concentrations (IC_50_) of antigen in solution with half-maximal binding (18). This assay was used to assess the solution interaction of these binding pairs where immobilized antigens and immune complexes formed on the surface are for readout only. VLPs were passively adsorbed to a high-binding microtiter plate at a fixed concentration (1.0 μg/mL, 100 μL/well). Two antigens were two-fold diluted from 2^0^ to 2^13^ with a starting concentration of 1.0 mg/mL. A constant amount of the anti-FMDV antibody was then added to the solution of the Ag dilution series, and the plate was incubated for 60 min. After washing, the horseradish peroxidase (HRP)–conjugated goat anti-mouse antibody (GAM-HRP) (Thermo Scientific, USA) was used for the quantitative detection of immune complexes formed by antibodies and antigens. TMB was used as the substrate, and the optical density of 450 nm was measured upon acid quenching. Curve fitting and IC_50_ calculations were performed with GraphPad Prism 6.0.

### Assessment of Immunity

The purified VLPs (100 and 25 μg/dose per 2 mL) were emulsified with adjuvant ISA 206 (SEPPIC, France) using the R30A Electric (FLUKO, German) under sterile conditions and were stored at 2–8°C until use. A total of 20 pigs were divided into four groups (Table 1) and immunized by intramuscular injection. Group 1 was designed for the evaluation of a commercial inactivated serotype O vaccine. Groups 2 and 3 were used to assess the effect of VLPs single immunization with the doses of 100 and 25 μg, respectively. Group 4 with phosphate-buffered saline (PBS) inoculation was set as the control. Blood samples were collected preimmune and weekly up to 10 weeks postimmunization, and sera anti-FMDV antibody was analyzed by a commercial LPB-ELISA kit (Lanzhou Veterinary Research Institute, China). Peripheral blood mononuclear cells (PBMCs) were isolated from all the pigs in the four groups at 28 dpi by centrifugation in Ficoll-Paque Plus (GE Healthcare, USA) at RT for 30 min.

**Table 1:** Experimental design of VLP-based FMD immune efficacy studies.

**Group**	**Number of**	**Immunogen**	**Primary**	**Boost at 4 wpi**
	**pigs**	**(2.00 mL/dose)**	**(dose)**	**(dose)**
1	5 (1#-5#)	Inactivated serotype O vaccine	1.0	1.0
2	5 (6#-10#)	VLPs (100 μg)	1.0	1.0
3	5 (11#-15#)	VLPs (25 μg)	1.0	1.0
4	5 (16#-20#)	PBS	1.0	1.0

### ELISpot Assays

FMDV-specific interferon γ (IFN-γ) SC frequencies were quantified to evaluate immune cell activation in splenocytes by porcine IFN-γ single-color enzymatic ELISpot assays (Cellular Technology Limited, USA). The plates were coated with 80 μL/well porcine IFN-γ capture solution at 4°C overnight. After washing with PBS one time, VLPs (1.0 and 10.0 μg/well) plus mitogen were plated in the wells (100 μL/well) and incubated in a 5% CO_2_ incubator for 20 min (performed in duplicate). After that, 100 μL of CTL-Test medium containing 3 × 10^5^ PBMCs was added to each well and then incubated at 37°C in a 5% CO_2_ incubator for 24 h. The concanavalin A (50 μg/mL, 100 μL/well; Sigma–Aldrich, USA) and PBS were used as the positive and negative controls, respectively. After treatment with 80 μL/well of antiporcine IFN-γ detection solution at RT for 2 h, the wells were incubated with 80 μL/well of tertiary solution for 30 min at RT and then with the addition of 80 μL/well of blue developer solution in the dark for 15 min at RT. Spot numbers were determined with a camera and analyzed using ImmunoSpot Software (Cellular Technology Limited, USA). The number of spots in negative controls was subtracted from counts of spot-forming cells in stimulated wells.

### Statistical Analyses

A one-way analysis of variance (ANOVA) test was applied to state the differences in antibody titers (different time points) and IFN-positive cell frequency (different concentrations of the VLPs during stimulation) among the three groups (inactivated vaccines, 25 μg VLP, 100 μg VLP).

## Results

### Expression of FMDV Capsid Proteins

**Figure 1:**
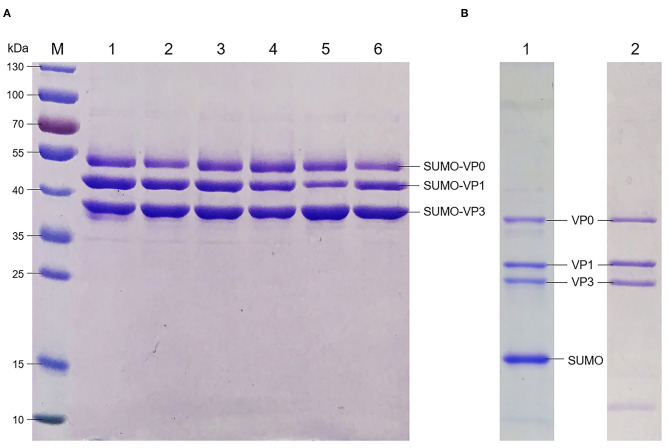
Identification of FMDV capsid protein expression, purification and SUMO cleavage. **(A)** Analysis of the FMDV capsid protein expression in *E. coli* with different permutation after purification with Ni^2+^ resin. M: protein marker; lanes 1–6: the recombinant proteins of Mya98-VP103, -VP130, -VP013, -VP031, -VP301, and -VP310. **(B)** SDS-PAGE analysis of Mya98-VP031 after SUMO removal. Lane 1: the protein treated with Ulp 1 protease; lane 2: the proteins (from the sample of line 1) purified by Ni^2+^ resin.

Six recombinant plasmids containing the tandem full-length of VP0, VP3, and VP1 genes in different permutations were constructed and transformed into BL21 (DE3). The protein expression was induced at 28°C by Isopropyl β-D-Thiogalactoside (IPTG). For accurate comparison of VP0, VP3, and VP1 expression levels in all the six tandem types, extracts original from all the transfected cells were purified by the Ni Sepharose 6 Fast Flow for capturing the His ×6-fusion proteins. The quantification of the three SUMO fusion proteins was analyzed by Alpha Imager 2200; the results showed the proportion of three SUMO-fused proteins yield by pET-Mya98-VP031 closer to 1:1:1 than the other tandem types after purification (Figure 1A, Table 2).

**Table 2:** The relative proportion of the SUMO-fused proteins in supernatant after purification.

**Plasmids**	**Proportion of recombinant proteins (%)**		
	**VP0**	**VP1**	**VP3**
pET-Mya98-VP103	24.3	37.6	37.9
pET-Mya98-VP130	27.8	29.9	42.3
pET-Mya98-VP013	33.2	27.2	39.6
pET-Mya98-VP031	32.2	32.3	35.5
pET-Mya98-VP301	32.8	18.7	48.5
pET-Mya98-VP310	26.9	28.8	44.3

*The relative proportion of the three SUMO fusion proteins were analysis by Alpha Imager 2200*.

### VLPs Assembly *in vitro*

After treatment with different SUMO protease activity units at 4°C, the cleavage products remained water-soluble with their molecular weights about 38 kDa (VP0), 28 kDa (VP1), 25 kDa (VP3), and the SUMO tag of 15 kDa, respectively (Figure 1B, lane 1). Later, the capsid proteins were obtained by further purification of the digestion products by Ni^2+^ resin (Figure 1B, lane 2) prior to being kept in buffer A.

### Characterization of FMDV VLPs

**Figure 2:**
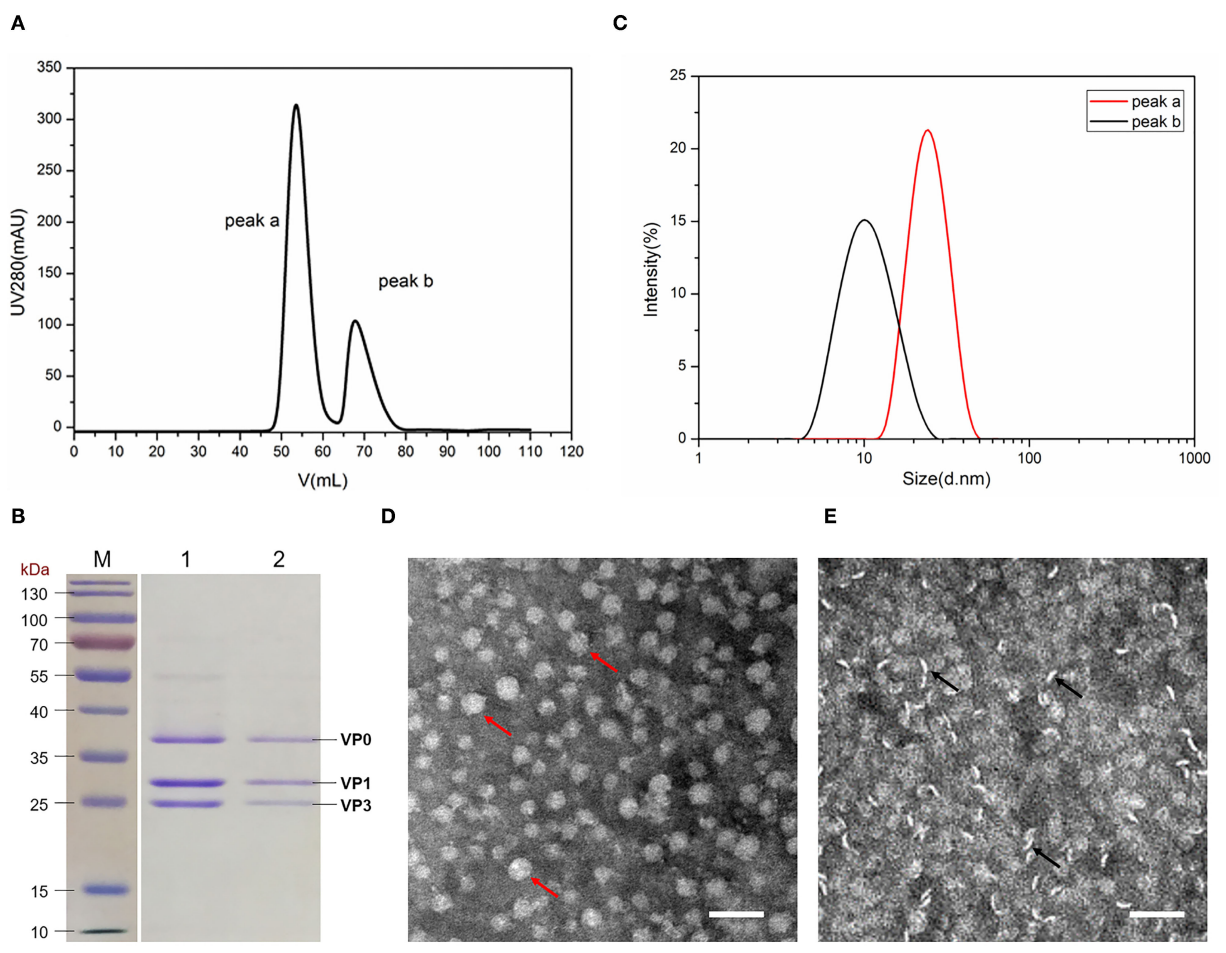
Characterization of FMDV VLPs. **(A)** Protein purification by gel filtration chromatography. **(B)** SDS-PAGE analysis of peak a, and peak b proteins obtained from gel filtration chromatography. Lane 1: peak a; lane 2: peak b. **(C)** DLS analysis of the size distribution of proteins in peaks a and b. **(D)** Protein visualization of peak a by TEM, the red arrow indicating the VLPs. **(E)** Protein visualization of peak B by TEM; the black arrow indicated the unassembled proteins. Magnification of 42,000×, the scale bar indicates 50 nm.

To demonstrate the efficiency and quality of the three FMDV proteins assembled into VLPs, the proteins aforementioned in buffer A were injected into the HiLoad 16/600 Superdex 200 pg gel filtration. The purified proteins eluted from the gel filtration column at the elution volume of 55 mL (peak a) and 70 mL (peak b) (Figure 2A), respectively.

Fractions that eluted from two monodisperse peak were analyzed by SDS-PAGE and DLS. As shown in Figure 2B, both peaks a and b contained VP0, VP1, and VP3 with a ratio of 1:1:1. Results of DLS showed the proteins in peak a with the diameter of 28 nm, suggesting VLPs formation, whereas the diameter of the protein in peak b was 10 nm, which might be the pentamer (Figure 2C). The diameters of VLPs in peak a were 20–30 nm with regularity and homogeneity after visualization by TEM (Figure 2D). The proteins in peak b were rod-shaped (Figure 2E), indicating that it was not assembled into VLPs.

### Effect of SUMO Cleaving Rate on VLPs Assembly *in vitro*

**Figure 3:**
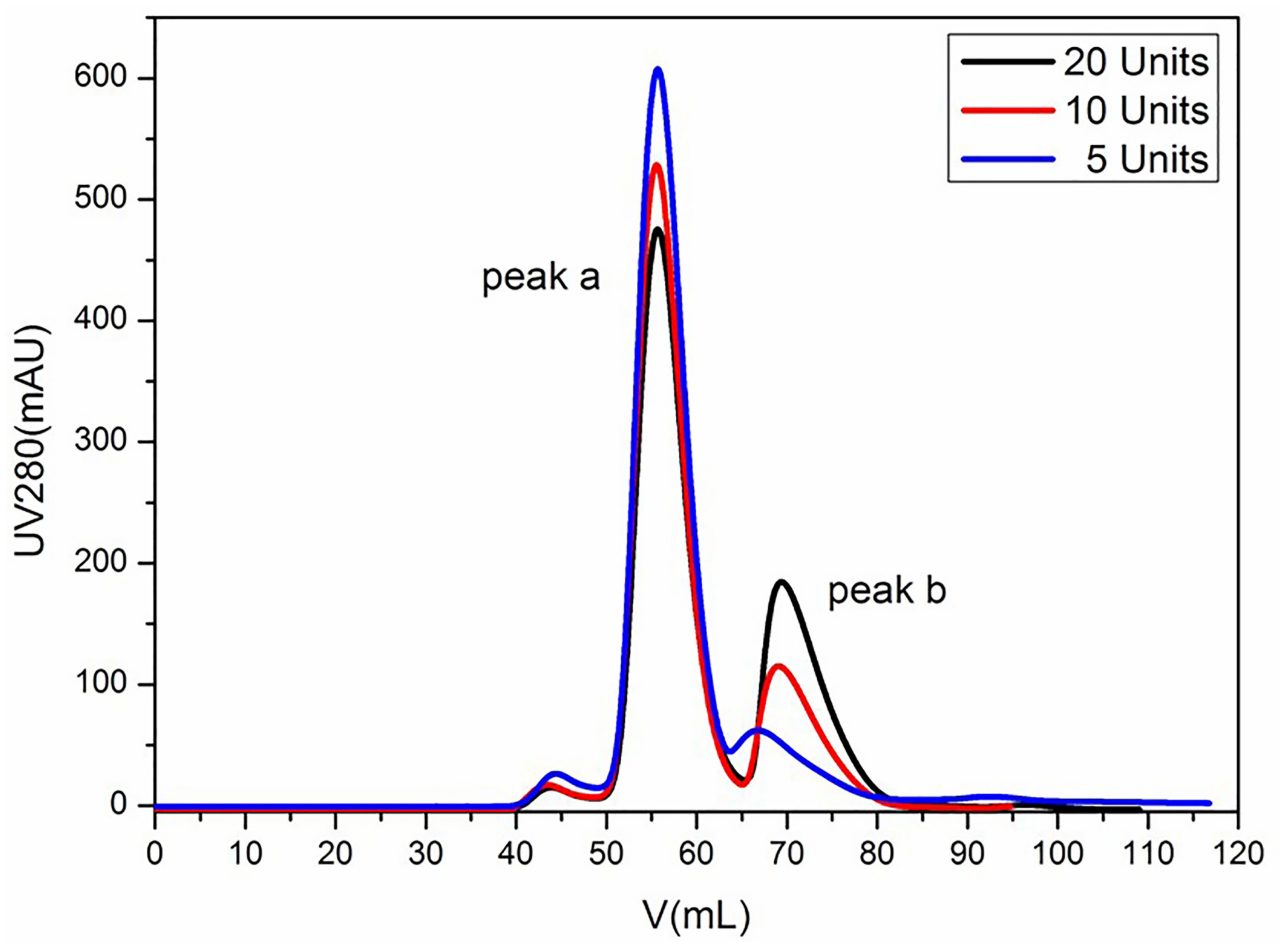
Analysis of FMDV VLPs by gel filtration chromatography. Lines in black, red, and blue represent the SUMO protease activity of 20, 10, and 5 units, respectively.

To further investigate the impact of SUMO cleaving activity on VLP assembly, the protein complex was treated with SUMO protease (5, 10, and 20 units, respectively) at 4°C until the His-SUMO was cleaved from the VP proteins completely. We found that the protease of 20, 10, and 5 units showed the complete cleavage effect in 5, 8, and 12 h, respectively (data not shown). After removing the His-SUMO tag by Ni Sepharose 6 Fast Flow, the proteins were analyzed using gel filtration chromatography. As shown in Figure 3, the distribution of peaks a and b varied with different protease conditions. The lower content of protease yielded the higher cluster of peak a, indicating that a tender activity of enzyme digestion favored the VLP assembly.

### Quantitating the Changes in Different Epitopes Upon VLPs and Unassembled Proteins

In order to dissect the discrepancies of individual epitopes on the VLPs and unassembled proteins, a solution competitive ELISA was developed. Four neutralizing mAbs of FMDV, F21-48, F21-64, F21-58, and F21-41, used in the solution competitive ELISA were previously studied (17), which recognized antigenic site 1 on the G-H loop of VP1 at Amino Acid (aa) 148, and aa 136–151, antigenic site 2 in the region of VP2 at aa 77, and antigenic site 3 of VP1 at aa 43–44, respectively.

**Figure 4:**
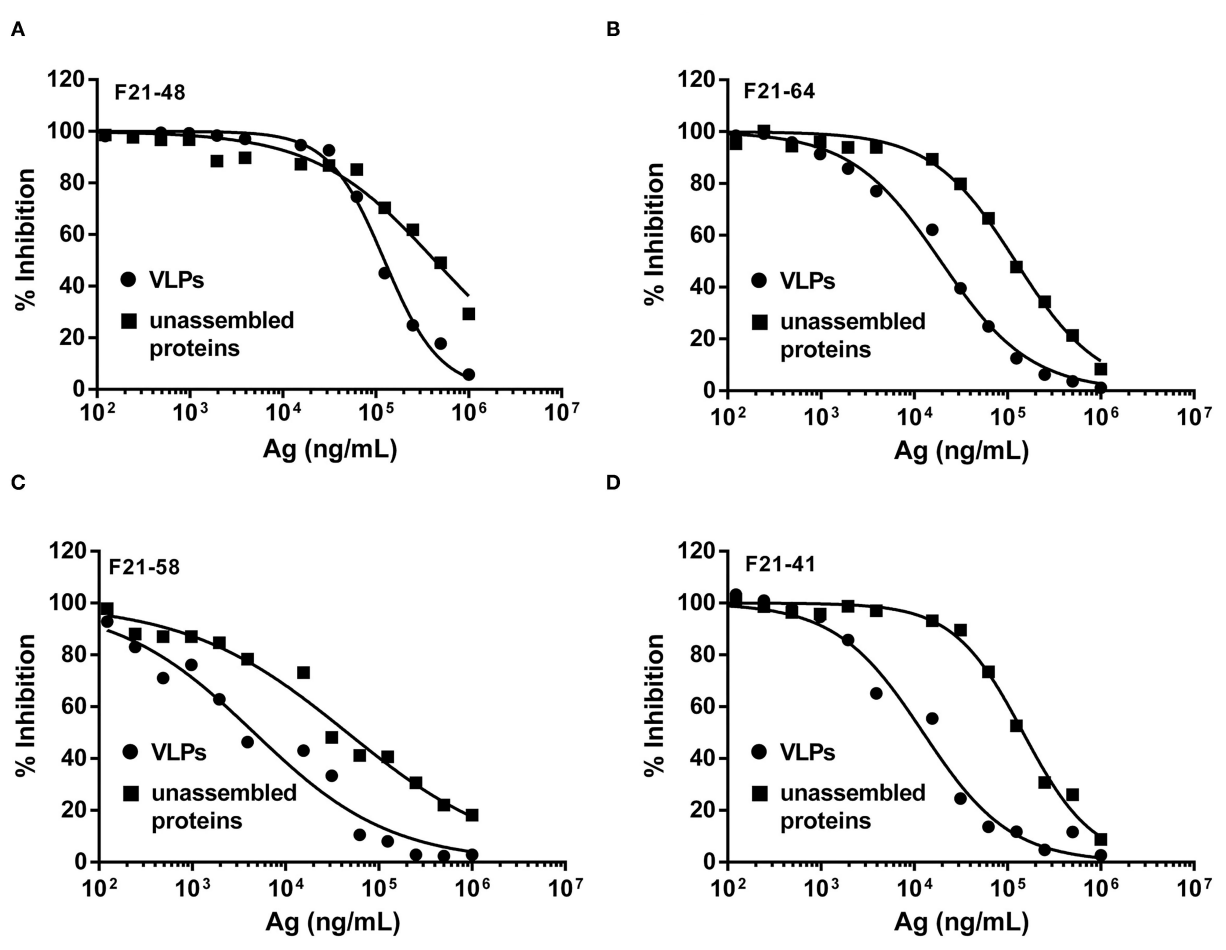
The solution competitive ELISA methods for quantitative antigenicity analysis of VLPs and unassembled proteins. The inhibition ability of mAbs F21-48, F21-64, F21-58, and F21-41 to VLPs (●) and unassembled proteins (■) were shown in **(A–D)**, respectively.

As shown in Figure 4 and Table 3, the binding behaviors of mAb (F21-48, F21-64, F21-58, and F21-41) to VLPs and unassembled proteins showed the inhibition profiles effect in the competitive ELISA. The IC_50_ values were derived and listed in Table 3. Significant improvements in the IC_50_ (3.5- to 12-folds) of the epitopes in VLPs for the neutralizing mAbs F21-48, F21-64, F21-58, and F21-41 were observed, which indicated that the higher antigenicity of VLPs than unassembled proteins.

**Table 3:** Quantitative analysis of two analogous FMDV proteins by the solution competitive ELISA concerning their binding profiles with different monoclonal antibodies.

**Neutralizing mAbs**	**IC**_****50****_ **(ng/mL)**		**Relative binding activity (VLPs over unassembled)**
	**Unassembled**	**VLPs**	
F21-64 (Site1a, 5)	121,754	19,245	6.3
F21-48 (Site1a, 5)	430,916	123,724	3.5
F21-41 (Site2)	146,927	12,449	12
F21-58 (Site3)	45,888	4,598	10

### Humoral- and Cell-Mediated Immune Responses in Pigs Elicited by FMDV VLPs

**Figure 5:**
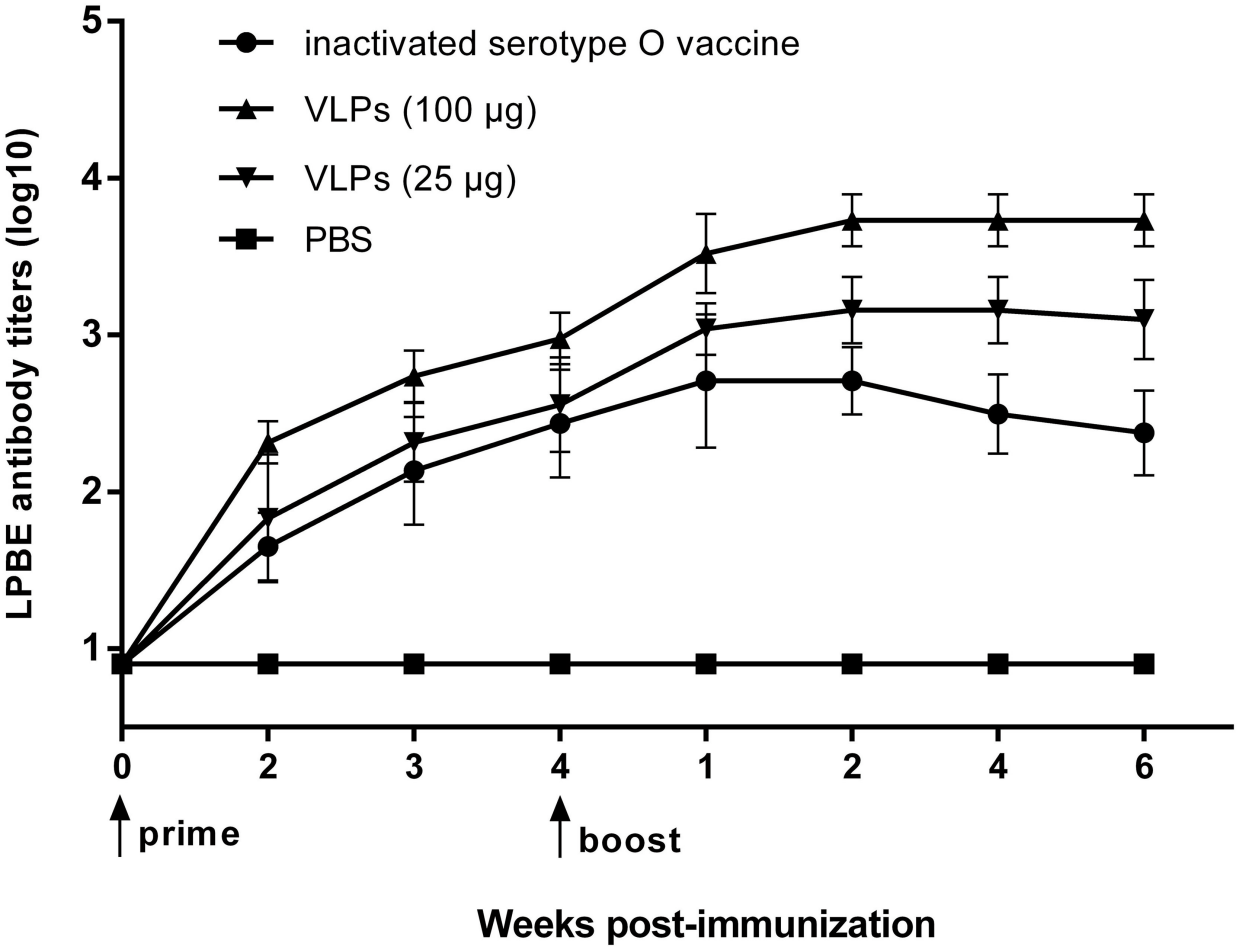
FMDV-specific immune responses in pigs. FMD immune efficacy studies. Pigs (*n* = 5) in three groups were immunized with inactivated FMDV serotype O vaccine (●) or FMD VLPs of 25 (▼) and 100 (▲) μg/dose, and five PBS-inoculated pigs in a control group (■). Serum samples were collected preimmune and weekly up to 10 weeks postimmunization for FMDV-specific antibody response detected by LPBE. The LPBE titers of PBS-inoculated pigs were all lower than 1:8. Data are expressed as the mean ± standard deviation (SD). The one-way ANOVA test was applied to state the differences on antibody titers among the three groups in each time point after immunization.

To determine the VLP immunity for stimulating anti-FMDV immune responses in pigs, three groups of pigs were immunized with inactivated FMDV vaccine or FMDV VLPs, and one group was inoculated with PBS as a negative control. As shown in Figure 5, the antibody titers of PBS-inoculated pigs were all negative during the entire experiment, and the antibody titers of the pigs in the group immunized with FMDV VLPs at the dose of 100 μg were higher than those in the group with the immune dose of 25 μg, and pigs in both of the VLPs immunized groups showed a higher antibody level than that in the inactivated FMDV vaccine-immunized group. All the data of antibody titers after immunization (different time points) showed a statistically significant difference (*p* < 0.001) in the one-way ANOVA.

**Figure 6:**
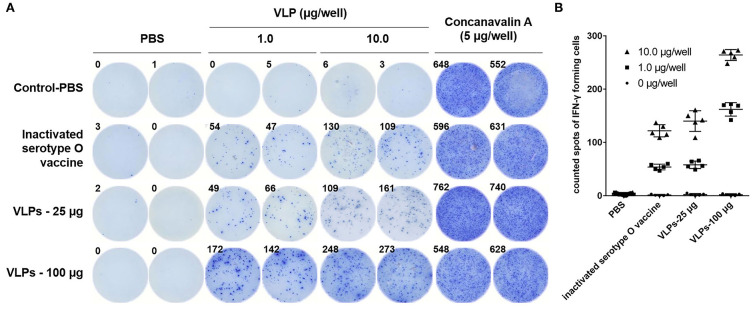
ELISpot assays of FMDV serotype O–specific IFN-γ after immunization with VLPs. **(A)** Representative IFN-γ ELISpot wells for the PBS, inactivated serotype O vaccine, and FMDV VLPs (25 and 100 μg/dose). The numbers in the upper left corners indicate counted spots of IFN-γ forming cells per 3 × 10^5^ responder PBMCs at 24 h after stimulation. **(B)** FMDV serotype O–specific IFN-γ of all immunized pigs; the concentrations of VLPs for stimulation were 0 μg/well (●), 1.0 μg/well (■), and 1.0 μg/well (▲). The PBMCs were collected at 28 dpi. Data are expressed as the mean ± standard deviation (SD). The one-way ANOVA test was applied to state the differences on IFN-positive cell frequency among the three groups (different concentrations of the VLPs during stimulation).

The FMDV-specific cellular immune response induced by VLPs was characterized by measuring IFN-γ SC responsiveness in PBMCs stimulated with FMDV VLPs *in vitro*. Compared with the negative control group, PBMCs from pigs with FMDV VLPs immunization triggered stronger cell activation for IFN-γ induction than in the inactivated FMDV vaccine immunized group, with dose-dependent positive correlations (Figures 6A,B), which correlated well with the antibody test results. The data of IFN-γ SC frequency (different concentrations of the VLPs during stimulation) showed a statistically significant difference (*p* < 0.001) in the one-way ANOVA.

Taken together, these results showed that FMDV VLP was an efficient antigen for eliciting both humoral and cell-mediated immune responses against FMDV.

## Discussion

In previous reports, SUMO-fused FMDV capsid proteins (VP0, VP1, and VP3) coexpressed by two or three plasmids in *E. coli* with multiple antibiotic selections were capable for the formation of the stable heterotrimeric complex (8, 9). However, the use of multiple plasmids and antibiotic selections may cause the imbalance of the capsid proteins in *E. coli* by obliteration of any plasmid. In our previous studies, three capsid proteins of FMDV serotype Asia 1 were coexpressed by a single plasmid (11). The possible interactions had been certified among the capsid proteins existence during the procession of its expression or assembly (19). So, the unequal expression level of three SUMO-fused proteins in this study tandemly in different permutations in the same plasmid may be influenced by the specific protein–protein interactions, and the optimal tandem permutation of VP0–VP3–VP1 may facilitate the formation of FMDV capsomer, which was coordinated to the capsid proteins in the precursor of FMDV P1. The efficient and uniform expression of the three capsid proteins probably resorted to their parallel tandem status as the veritable virus, but the mechanism therein remained to be blurry at present.

Myristoylation, which did not exist in the prokaryotic system, is important for the assembly of virus capsids (5, 20), while the VLPs of FMDV could be produced correctly in prokaryotic cells (8, 11); this may be due to the influence of other factors such as static electricity and hydrophobicity, which should be studied in the future. In this study, the SUMO tag well advanced the protein production level as well as the solubility profile of overexpressed proteins, while the interactions of the three SUMO-fused proteins were different compared with those of naive virus, the self-assembly of VLPs was still primarily based on the SUMO tag excision (9). Previous studies showed limited attentions to the effect of SUMO tag dislodging on the VLP assembly process (8, 9, 11), but with no detail of the regulation of VLP assembly and epitope exposure in addition to the effectiveness of VLPs illustrated by animal experiments. Here, the VLP antigen with the best immunogenicity was obtained after the evaluation of the regulation of VLP assembly and epitope exposure. In this study, it was sustained that a lower cracking rate of the SUMO tag was more conducive to FMDV VLP assembly bearing a correct spatial conformation, which rendered the speculation that the quality and stability of VLPs can be improved through enzymolysis condition optimization.

The results of solution competitive ELISA (17) using four neutralizing mAbs for VLPs quantitative analysis showed that the VLP surface might contain multiple neutralizing epitopes after valid assembly, while the improper assembly complex preserved an inferior antibody titer, which conformed by TEM. The neutralizing antigenic sites of FMDV in different serotypes had been described with critical residues determined by MAb escape mutant studies (21–25). Five neutralizing antigenic sites had been identified on the surface of FMDV serotype O (26, 27). As the structural protein VP4 was entirely internal of the FMDV serotype O capsid, its neutralizing antigen sites were primarily located on the capsid proteins VP1-3 (28–30).

The VLPs capable of eliciting a protective immune response were more likely achieved by mimicking the authentic epitopes of FMDV virions. So, epitope-specific antigenicity was important for the understanding of the VLPs structures, particularly the presence of the neutralizing epitopes. Here, the exposure of multiple neutralization epitopes on the VLPs surface can stimulate the high level of humoral- and cell-mediated immune responses in pigs, indicating the high potential of immune protection of the VLPs.

In conclusion, in this study, the expression of FMDV capsid proteins in *E. coli* achieved the ratio of 1:1:1 by tandem permutation of VP0–VP3–VP1. Strict control of the formation process of VLPs at a low protease level can maximize the exposure of multiple neutralized epitopes, which is sufficient to induce FMDV-specific humoral and cell-mediated immune responses. It provides a stable and reliable candidate antigen for the development of the subunit vaccine.

## Data Availability Statement

The original contributions presented in the study are included in the article/Supplementary Material, further inquiries can be directed to the corresponding author/s.

## Ethics Statement

The animal study was reviewed and approved by Research Ethics Committee of the National Research Center for Veterinary Medicine.

## Author Contributions

YX, SZ, and WP conceived and designed the research. SZ, HY, XG, YW, XX, MW, and HZ conducted the experiments. MY contributed FMDV MAbs. YX, SZ, and WP analyzed the data. SZ, YX, BH, and KT conceived the study, carried out additional analyses, and finalized the manuscript. All authors contributed to the article and approved the submitted version.

## Conflict of Interest

The authors declare that the research was conducted in the absence of any commercial or financial relationships that could be construed as a potential conflict of interest.
