# Supplementary figures and images for: The High Immunity Induced by the Virus-Like Particles of Foot-and-Mouth Disease Virus Serotype O
Source: Front Vet Sci. 2021 Feb 25;8:633706. doi: 10.3389/fvets.2021.633706 (PMC7947224; doi:10.3389/fvets.2021.633706)

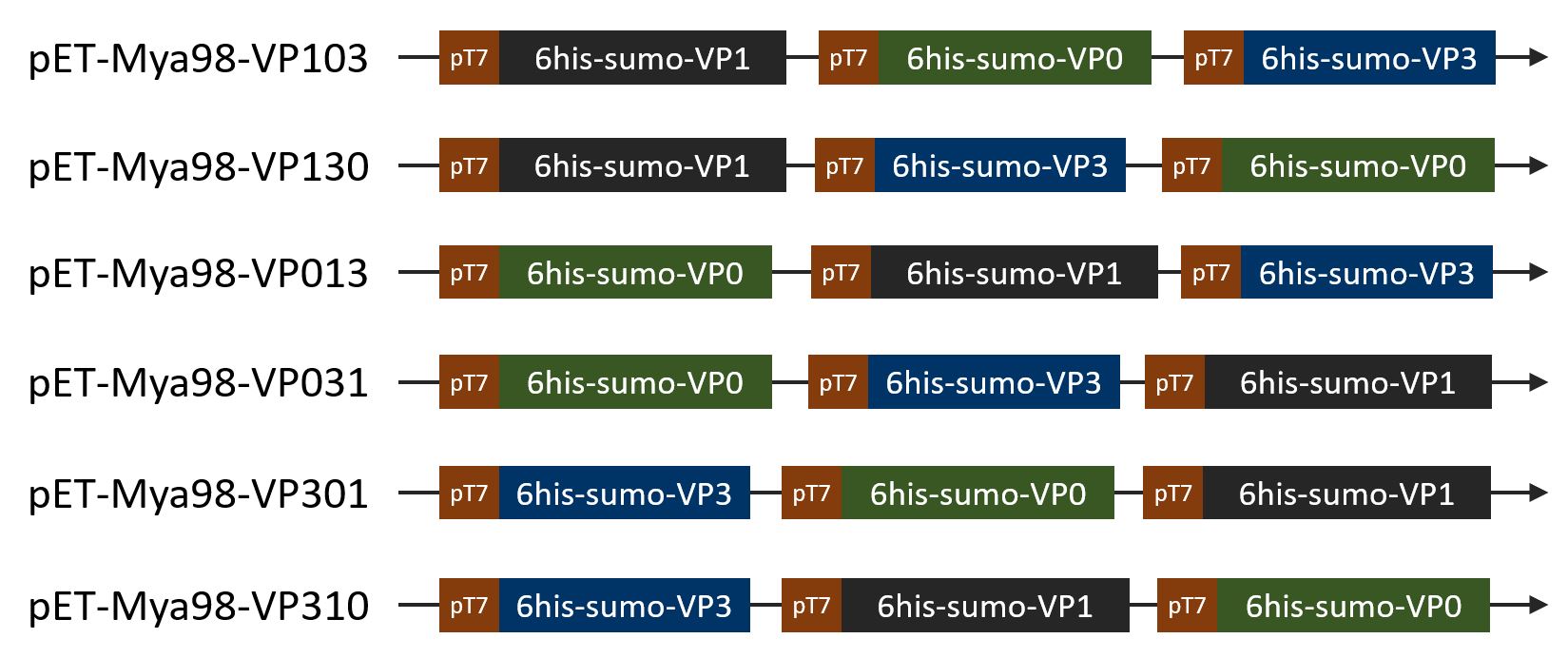

Supplement: Supplementary file 1 [file Image_1.JPEG]

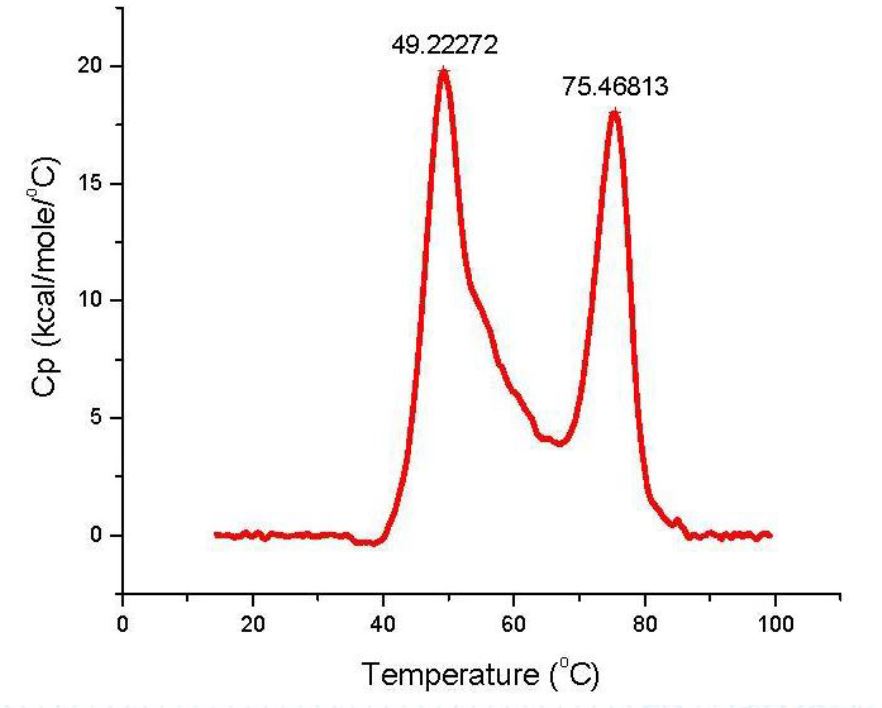

Supplement: Supplementary file 2 [file Image_2.JPEG]
