# Supplementary material for: The High Immunity Induced by the Virus-Like Particles of Foot-and-Mouth Disease Virus Serotype O
Source: Front Vet Sci. 2021 Feb 25;8:633706. doi: 10.3389/fvets.2021.633706 (PMC7947224; doi:10.3389/fvets.2021.633706)
Supplement: Supplementary file 3 [file Table_1.DOCX]

Supplementary Table 1

Primers list for plasmid construction.

| vectors | primer | sequence（5‘--3’） |
| --- | --- | --- |
| pET-Mya98 -VP031 | P1 | CATGCCATGGGCATGGGCAGCAGCCATCAT |
|  | P2 | GGAATTCCATATGTTATTCTTTAGACGGGAA |
|  | P3 | CGCGGATCCATCTCGATCCCGCGAAAT |
|  | P4 | CGAGCTCTTACTGCTGACGAGCGTCAA |
|  | P5 | GCGTCGACATCTCGATCCCGCGAAAT |
|  | P6 | ATAAGAATGCGGCCGCTTACAGAGACTGTTTAACCG |
| pET-Mya98 -VP013 | P1 | CATGCCATGGGCATGGGCAGCAGCCATCAT |
|  | P2 | GGAATTCCATATGTTATTCTTTAGACGGGAA |
|  | P3 | CGCGGATCCATCTCGATCCCGCGAAAT |
|  | P7 | CGAGCTCTTACAGAGACTGTTTAACCG |
|  | P5 | GCGTCGACATCTCGATCCCGCGAAAT |
|  | P8 | ATAAGAATGCGGCCGCTTACTGCTGACGAGCGTCAA |
| pET-Mya98 -VP103 | P1 | CATGCCATGGGCATGGGCAGCAGCCATCAT |
|  | P9 | GGAATTCCATATGTTACAGAGACTGTTTAACCG |
|  | P3 | CGCGGATCCATCTCGATCCCGCGAAAT |
|  | P10 | CGAGCTCTTATTCTTTAGACGGGAA |
|  | P5 | GCGTCGACATCTCGATCCCGCGAAAT |
|  | P8 | ATAAGAATGCGGCCGCTTACTGCTGACGAGCGTCAA |
| pET-Mya98 -VP130 | P1 | CATGCCATGGGCATGGGCAGCAGCCATCAT |
|  | P9 | GGAATTCCATATGTTACAGAGACTGTTTAACCG |
|  | P3 | CGCGGATCCATCTCGATCCCGCGAAAT |
|  | P4 | CGAGCTCTTACTGCTGACGAGCGTCAA |
|  | P5 | GCGTCGACATCTCGATCCCGCGAAAT |
|  | P11 | ATAAGAATGCGGCCGCTTATTCTTTAGACGGGAA |
| pET-Mya98 -VP310 | P1 | CATGCCATGGGCATGGGCAGCAGCCATCAT |
|  | P12 | GGAATTCCATATGTTACTGCTGACGAGCGTCAA |
|  | P3 | CGCGGATCCATCTCGATCCCGCGAAAT |
|  | P7 | CGAGCTCTTACAGAGACTGTTTAACCG |
|  | P5 | GCGTCGACATCTCGATCCCGCGAAAT |
|  | P11 | ATAAGAATGCGGCCGCTTATTCTTTAGACGGGAA |
| pET-Mya98 -VP301 | P1 | CATGCCATGGGCATGGGCAGCAGCCATCAT |
|  | P12 | GGAATTC GGATCC TTACTGCTGACGAGCGTCAA |
|  | P3 | CGCGGATCCATCTCGATCCCGCGAAAT |
|  | P10 | CGAGCTCTTATTCTTTAGACGGGAA |
|  | P5 | GCGTCGACATCTCGATCCCGCGAAAT |
|  | P6 | ATAAGAATGCGGCCGCTTACAGAGACTGTTTAACCG |

Note: Restriction sites are underlined
